# ARE YOU A REAL PARTICIPANT? EXPERIENCES WITH FRAUDULENT PARTICIPATION ACROSS TWO FUNDED STUDIES

**DOI:** 10.1093/geroni/igad104.1179

**Published:** 2023-12-21

**Authors:** Cara Wallace

**Affiliations:** Trudy Busch Valentine School of Nursing, Saint Louis University, St. Louis, Missouri, United States

## Abstract

While offering financial incentives for participation in research studies is common, less discussed is the potential impact of fraudulent participation for financial gain. Survey and data management software provide safeguards for fraud detection but enabling detection does not automatically weed out fraudulent responses. Few resources provide guidelines for identifying or managing fraudulent participation. Thoughtful processes are important, while also being mindful of normal research behaviors which might get flagged as suspicious, for example due to low literacy skills. This presentation discusses experiences across two studies with adults living with serious illness and/or their family members and two different types of fraudulent participation: online surveys and studies requiring personal interaction. Study one is a pilot instrument validation study collecting online survey data. Recruitment occurred locally through listservs and a research participation database from a large university hospital. Study two is a mixed-methods longitudinal design where survey and interview data are collected via phone or video calls. Participants for this study were recruited nationwide using multiple strategies, including social media. Both offer time-saving insights for dealing with fraudulent participation in research. For example, study one provides tips for planning how to manage spam/bots prior to data collection, including setting up filters to automatically weed out identified spam, creating automatic messaging included in the survey in the event it needs to be paused, and identifying potential problematic patterns in data that bypasses fraud detection. Study 2 emphasizes the importance of creating strong verification procedures for study eligibility when relying on participant reporting.

